# Mitochondrial DNA Damage and Brain Aging in Human Immunodeficiency Virus

**DOI:** 10.1093/cid/ciaa984

**Published:** 2020-07-28

**Authors:** Carla Roca-Bayerri, Fiona Robertson, Angela Pyle, Gavin Hudson, Brendan A I Payne

**Affiliations:** 1 Wellcome Centre for Mitochondrial Research, Translational and Clinical Research Institute, Newcastle University, Newcastle-upon-Tyne, United Kingdom; 2 Wellcome Centre for Mitochondrial Research, Biosciences Institute, Newcastle University, Newcastle-upon-Tyne, United Kingdom; 3 Department of Infection and Tropical Medicine, Newcastle-upon-Tyne Hospitals, Newcastle-upon-Tyne, United Kingdom

**Keywords:** mitochondrial DNA, HIV, antiretroviral therapy, HIV-associated neurocognitive disorders, aging

## Abstract

**Background:**

Neurocognitive impairment (NCI) remains common in people living with human immunodeficiency virus (PLWH), despite suppressive antiretroviral therapy (ART), but the reasons remain incompletely understood. Mitochondrial dysfunction is a hallmark of aging and of neurodegenerative diseases. We hypothesized that human immunodeficiency virus (HIV) or ART may lead to mitochondrial abnormalities in the brain, thus contributing to NCI.

**Methods:**

We studied postmortem frozen brain samples from 52 PLWH and 40 HIV-negative controls. Cellular mitochondrial DNA (mtDNA) content and levels of large-scale mtDNA deletions were measured by real-time polymerase chain reaction. Heteroplasmic mtDNA point mutations were quantified by deep sequencing (Illumina). Neurocognitive data were taken within 48 months antemortem.

**Results:**

We observed a decrease in mtDNA content, an increase in the mtDNA “common deletion,” and an increase in mtDNA point mutations with age (all *P* < .05). Each of these changes was exacerbated in HIV-positive cases compared with HIV-negative controls (all *P* < .05). ART exposures, including nucleoside analogue reverse transcriptase inhibitors, were not associated with changes in mtDNA. The number of mtDNA point mutations was associated with low CD4/CD8 ratio (*P* = .04) and with NCI (global T-score, *P* = .007).

**Conclusions:**

In people with predominantly advanced HIV infection, there is exacerbation of age-associated mtDNA damage. This change is driven by HIV per se rather than by ART toxicity and may contribute to NCI. These data suggest that mitochondrial dysfunction may be a mediator of adverse aging phenotypes in PLWH.

## Teaser

Brain tissue from people living with HIV shows depletion of mitochondrial DNA (mtDNA) content and an increase in mtDNA mutations. These changes augment those seen with normal aging and may contribute to cognitive impairment.

**(See the Editorial Commentary by Hulgan and Samuels on pages e474–6.)**

Neurocognitive impairment (NCI) remains a prevalent complication of human immunodeficiency virus (HIV) infection despite the widespread availability of combination antiretroviral therapy (cART) [1]. A large US study suggested that about half of people living with HIV (PLWH) have some detectable NCI according to the Frascati criteria for HIV-associated neurocognitive disorders [2]. The reasons for this persistence of NCI in PLWH remain incompletely understood, but are likely to be multifactorial and may plausibly include a “legacy” effect of neuronal damage prior to initiation of cART and persistent microglial activation [3]. In contrast, the possible contribution of cART toxicity is unclear and published data are conflicting [4, 5].

Acquired mitochondrial dysfunction has been implicated in many degenerative brain diseases, as well as in the normal aging process [6]. Mitochondria contain their own DNA (mtDNA), which is present in many hundreds to thousands of genome copies per cell, and is especially abundant in cells with high energy demand such as neurons. Typical mtDNA changes seen in brain aging and in conditions such as Alzheimer disease and Parkinson disease include a decrease in mtDNA content (depletion) and an increase in somatic mtDNA mutations (both large-scale deletions and point mutations) [7–9].

Mitochondrial dysfunction and mtDNA damage are also well-recognized in PLWH. These phenomena have been considered largely as complications of antiretroviral therapy (ART), especially the nucleoside analogue reverse transcriptase inhibitor (NRTI) class [10]. While NRTIs principally inhibit HIV reverse transcriptase, they may also inhibit the mtDNA polymerase, pol γ, leading to mtDNA depletion [11]. More recently it has been demonstrated that NRTI therapy may also result in an increase in somatic mtDNA mutations in several tissues including skeletal muscle, blood, and the renal tract [12–15]. However, there is also some limited evidence that HIV per se may also reduce mtDNA content, at least in lymphocytes [16].

It is therefore plausible that HIV infection and/or ART is associated with an excess of mtDNA damage in the brain. One previous magnetic resonance spectroscopy study has suggested mitochondrial dysfunction in the frontal lobe in association with NRTI exposure, providing indirect support for this hypothesis [17]. One recent study on human brain tissue detected mtDNA defects, but these seemed to be mediated by exposure to drugs of abuse (methamphetamine), rather than HIV or ART factors [18]. Finally, a number of rodent studies have suggested that certain NRTIs may induce mitochondrial toxicity in the brain [19].

We therefore aimed to determine whether HIV or ART is associated with mtDNA changes in the brain of PLWH, and whether this potentially contributes to NCI.

## MATERIALS AND METHODS

### Samples

Snap-frozen postmortem frontal gray matter samples were obtained from the National NeuroAIDS Tissue Consortium (NNTC) [20], the Edinburgh Brain and Tissue Bank (EBTB) [21], and the Newcastle Brain Tissue Resource (NBTR) [22]. In light of the known associations between mtDNA changes and age, we specifically included HIV-negative controls of comparable age range to the HIV-positive cases, but also older controls to determine whether the effects of HIV/ART mirrored those seen in normal aging. Donors with other (non-HIV-associated) brain pathology or opportunistic infections of the brain were excluded. Maximum postmortem interval was 24 hours. Clinical and neurocognitive testing data were taken from the last antemortem study visit (maximum 48 months antemortem). Brain bank donors had given informed consent for retention of their tissue for research purposes, and the present study was approved by the local research ethics committee.

### Molecular Assays

Primers and probes were purchased from Integrated DNA Technologies (Leuven, Belgium). Primer and probe sequences are shown in Supplementary Table 1. The mtDNA content and proportional levels of the δ4977 bp mtDNA “common deletion” (CD) were simultaneously measured by triplex real-time quantitative polymerase chain reaction (qPCR) assay in a 20-µL reaction comprising 1x iTaq Universal Probes Supermix (Bio-Rad), 300 nM of each forward and reverse *b2M* and CD primers, 75 nM of each forward and reverse *MT-ND1* primers, 200 nM of each probe, with 2 µL of DNA. The qPCR conditions were 95°C for 3 minutes; followed by 40 cycles of 95°C for 10 seconds and 62°C for 1 minute. The mtDNA content was expressed as mtDNA copies per cell, based on simultaneous quantification of the mtDNA and the nuclear DNA targets. CD levels were expressed as the proportion of mtDNA molecules that contained the CD mutation. Proportional CD levels were expressed on a log_10_ scale in keeping with the distribution of the data.

Long-range PCR (LR-PCR) was used to screen for large-scale mtDNA deletions. LR-PCR was performed in 25 μL reactions comprising 1× PrimeSTAR GXL Buffer Mg_2_+ (Takara Bio), 200 nM dNTPs, 200 nM primers (“fragment 1”), 0.625 U PrimeSTAR GXL DNA polymerase (Takara Bio), and 1 μL DNA. PCR conditions consisted of 94°C for 1 minute, followed by 35 cycles of 98°C for 10 seconds, 60°C for 15 seconds, and 68°C for 10 minutes, with a final extension at 72°C for 10 minutes. Large-scale deletions were classified as present or absent in each sample based on visualization of the agarose gel image of LR-PCR products.

Two overlapping LR-PCR amplicons (“fragment 1” and “fragment 2”) were used to enrich mtDNA for deep sequencing, while avoiding nuclear pseudogene amplification. Library preparation was performed by Nextera XT Kit (Illumina) according to the manufacturer’s protocols. Multiplexed pools were sequenced using MiSeq version 3.0 sequencing kit (Illumina) in 250-bp paired-end reads. Case and control samples were randomly distributed across sequencing runs.

### Bioinformatic Analysis of MiSeq Data

MiSeq data were analyzed by a custom pipeline scripted by author F. R. The fastq files were first assessed for quality via FASTQC version 0.11.5 [23], before duplicate reads were removed by Fastuniq version 1.1 [24]. De-duplicated read sets were aligned using BWA, version 0.7.15, invoking the mem module [25], against genome versions GRCh37 (GCA_000001405.1). Sorting and indexing of alignments was performed using Samtools version 1.3.1 [26]. Variant calling was preformed using VarScan version 2.3.7 [27], and annotated via ENSEMBL VEP version 90 [28]. Samples were removed if coverage was <99% at a minimal depth of 1000×. Variants detected at >98% frequency were considered as homoplasmic, and variants at 2%–98% frequency as heteroplasmic. Variants at <2% heteroplasmy are not reported. When considering heteroplasmy levels of detected variants, we adjusted these variant frequencies relative to the assumed consensus mtDNA sequence for that subject. Thus, variants detected at 50%–98% heteroplasmy levels were assumed to represent somatic mutation away from a nonreference base position. We expressed “mutation count” as the number of mtDNA base positions in a given sample that showed variants. For the measure of “mutation load,” we weighted each variant base position by its mutation frequency (heteroplasmy level) before summing these variants and dividing by the number of base positions.

### Neurocognitive Analyses

Neurocognitive data were provided by NNTC and had been previously collected according to its standardized protocols, including appropriate adjustments for educational level and language [29]. (Neurocognitive data were not available for NBTR or EBTB subjects.) Primary neurocognitive diagnosis was categorized in accordance with the Frascati criteria [2]. We additionally report global T-score, global deficit score (GDS), and abstraction executive domain deficit score. Executive function was specifically studied owing to its anatomical correlation with the study samples (frontal lobe).

### Statistical Analyses

Statistical testing was performed using SPSS version 23 software (IBM SPSS, Armonk, New York). Log_10_ transformation of CD values was used to allow parametric testing. The relationships between age and mtDNA content or CD levels were examined by linear regression. Generalized linear modeling (negative binomial distribution with log link) was used for analyses of mtDNA point variants.

Given the large literature linking changes in mtDNA with age, all analyses of mtDNA content were corrected for age at death. This correction was applied by first calculating the regression coefficient (B) for age in our HIV-negative control subjects (B = −5.512 mtDNA copies per year), then applying a correction of age × B. We applied a similar age correction for relative CD levels (B = .0158 per year).

## RESULTS

We studied a total of 92 donors (52 HIV positive, 40 HIV negative). Donor demographic and clinical characteristics are detailed in Table 1.

**Table 1.** Clinical and Neurocognitive Characteristics of the Study Cohort

Characteristic	HIV Positive	HIV Negative
No. of cases	52	40
Male sex	85%	70%
Age, y, mean (range)	49 (32–70)	66 (29–91)
On ART at last antemortem study visit	74%	…
Ever ART treated	93%	…
Dideoxy-NRTI (“d-drug”) exposed	58%	…
HIV-1 plasma RNA <400 copies/mL	27%	…
Current CD4 count, cells/µL, median (IQR)	71 (25–203)	…
Nadir CD4 count, cells/µL, median (IQR)	69 (12–99)	…
CD4:CD8 ratio, median (IQR)	0.14 (0.06–0.33)	…
HAND category (NN, ANI, MND, HAD)	9, 6, 14, 11	…
Global T-score, mean (SD)	43.9 (6.9)	…
Global deficit score, median (IQR)	0.4 (0.2–1.0)	…
Global clinical rating, median (IQR)	5 (4–6)	…

Abbreviations: ANI, asymptomatic neurocognitive impairment; ART, antiretroviral therapy; HAD, human immunodeficiency virus–associated dementia; HAND, human immunodeficiency virus–associated neurocognitive disorders; HIV, human immunodeficiency virus; IQR, interquartile range; MND, mild neurocognitive disorder; NN, neurocognitively normal; NRTI, nucleoside reverse transcriptase inhibitor.

### Cellular mtDNA Content

**Figure 1.**
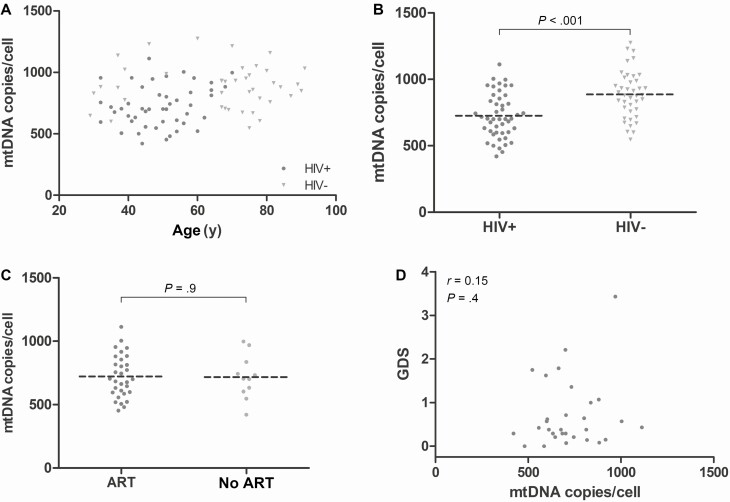
Mitochondrial DNA (mtDNA) content in frontal cortex samples. *A*, The mtDNA content decreased with age. *B*, After correction for age, mtDNA content was lower in human immunodeficiency virus (HIV)–positive cases than controls. Among HIV-positive cases, mtDNA content did not differ according to antiretroviral therapy treatment status at last visit (*C*) or cognitive function (*D*). Abbreviations: ART, antiretroviral therapy; GDS, global deficit score; HIV, human immunodeficiency virus; mtDNA, mitochondrial DNA.

We first determined whether HIV status affected mtDNA content. Mean mtDNA content in HIV-positive cases was 726/cell (standard error [SE], 24; n = 47) compared with 887 (SE, 28; n = 40) in HIV-negative controls (*P* < .001; Figure 1A and 1B).

Next we investigated the effect of HIV clinical and treatment factors on mtDNA content among HIV-positive cases. There were no significant correlations between mtDNA content and immunological parameters or HIV viral load: CD4 lymphocyte count (Pearson regression coefficient [*r*] = 0.14, n = 44, *P* = .4); CD4/CD8 ratio (*r* = −0.11, n = 23, *P* = .6); log_10_ HIV-1 RNA plasma viral load (*r* = −0.22, n = 44, *P* = .14) (Supplementary Figure 1*A*–*C*). Furthermore, there was no association between ART and mtDNA content, based either on current exposure to any ART (ART: 722 [30], n = 31; no ART: 717 [52], n = 11; *P* = .9), or history of exposure to 1 or more of those NRTIs that most strongly inhibit mitochondrial pol γ in vitro (ie, the dideoxynucleoside analogues [d-drugs]: didanosine [ddI], zalcitabine [ddC], or stavudine [d4T]) (any d-drug exposure: 692 [35], n = 23; no d-drug exposure: 752 [40], n = 17; *P* = .27). The same was true for these NRTIs considered individually: ddI exposure (ddI: 694 [42], n = 18; no ddI: 737 [33], n = 22; *P* = .4); ddC exposure (ddC: 774 [59], n = 10; no ddC: 699 [29], n = 30; *P* = .2); or d4T exposure (d4T: 671 [35], n = 21; no d4T: 769 [37], n = 19; *P* = .06). Zidovudine (ZDV) is a relatively weak inhibitor of pol γ inhibition, but can affect mtDNA replication via other pathways. Like the d-drugs, ZDV exposure did not affect mtDNA content (ZDV: 743 [36], n = 23; no ZDV: 683 [38], n = 17; *P* = .3). We also examined nonnucleoside reverse transcriptase inhibitor (NNRTI) and protease inhibitor (PI) exposure, and neither was associated with mtDNA content (NNRTI: 656 [53], n = 11; no NNRTI: 741 [30], n = 29; *P* = .15; PI: 723 [36], n = 21; no PI: 712 [39], n = 19; *P* = .8) (Figure 1C, Supplementary Figure 1*D*–*I*).

We then investigated the association between mtDNA content and NCI. Based on the Frascati categorical classification of NCI (normal; asymptomatic neurocognitive impairment [ANI]; mild neurocognitive disorder [MND]; HIV-associated dementia [HAD]), no association was seen (1-way analysis of variance [ANOVA], *P* = .33). This was also true of global T-score (*r* = −0.20, n = 30, *P* = .28), and GDS (*r* = 0.15, n = 30, *P* = .4). In view of the anatomical site of tissue studied, we also specifically assessed executive function. Here we saw a trend toward association between deficit score for this cognitive domain and mtDNA content (*r* = 0.31, n = 33, *P* = .077) (Figure 1D, Supplementary Figure 1*J*–*L*). Verbal fluency deficit score was significantly associated with mtDNA content (*r* = −0.36, n = 34, *P* = .034), but no significant association was seen for any other cognitive domain.

### Large-scale mtDNA Deletions

**Figure 2.**
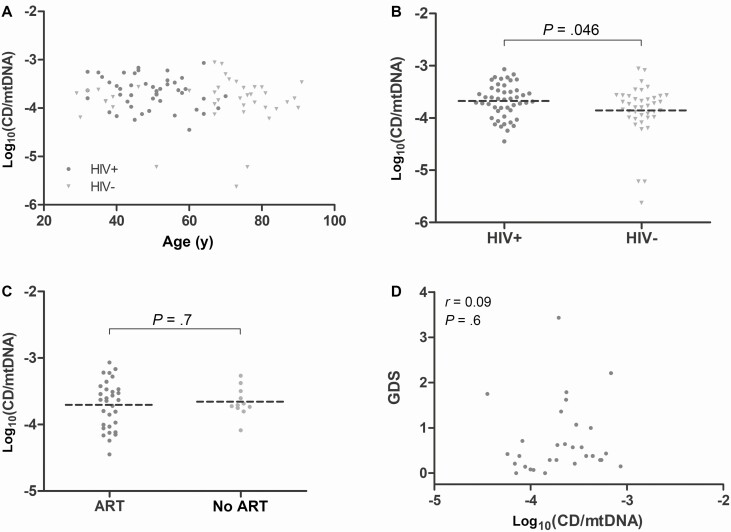
Proportional levels of the mitochondrial DNA common deletion (CD) in frontal cortex samples. *A*, CD levels increased with age. *B*, After correction for age, CD levels were higher in human immunodeficiency virus (HIV)–positive cases than controls. Among HIV-positive cases, CD levels did not differ according to antiretroviral therapy treatment status at last visit (*C*) or cognitive function (*D*). Abbreviations: ART, antiretroviral therapy; CD, common deletion; GDS, global deficit score; HIV, human immunodeficiency virus; mtDNA, mitochondrial DNA.

No large-scale mtDNA rearrangements were detectable by LR-PCR. We therefore proceeded to apply a highly sensitive CD-specific qPCR assay. Positive HIV status was associated with higher proportional levels of the CD. Mean (log_10_) CD in HIV-positive cases was −3.67 /mtDNA (SE, 0.05; n = 47) compared with −3.86 (SE, 0.08; n = 40) in HIV-negative controls (*P* = .046; Figure 2A and 2B).

Among HIV-positive cases, there were no significant correlations between mtDNA CD levels and CD4 lymphocyte count (*r* = −0.13, n = 44, *P* = .4); CD4/CD8 ratio (*r* = 0.05, n = 23, *P* = .8); or viral load (*r* = −0.06, n = 44, *P* = .7) (Supplementary Figure 2*A*–*C*). Furthermore, there was no association between ART and mtDNA CD levels (current ART: −3.70 [0.06], n = 31; no ART: −3.66 [0.07], n = 11; *P* = .7), or d-drug exposure (d-drugs: −3.68 [0.08], n = 23; no d-drugs: −3.66 [0.06], n = 17; *P* = .8). This was also true of the specific d-drugs and ZDV: ddI exposure (ddI: −3.67 [0.09], n = 18; no ddI, −3.68 [0.05]: n = 22; *P* = .9), ddC exposure (ddC: −3.65 [0.1], n = 10; no ddC: −3.68 [0.05], n = 30; *P* = .8), d4T exposure (d4T: −3.67 [0.08], n = 21; no d4T: −3.68 [0.06], n = 19; *P* = .9), ZDV exposure (ZDV: −3.6 [0.07], n = 23; no ZDV: −3.75 [0.08], n = 17; *P* = .18). Likewise, there was no association of CD levels with NNRTI or PI exposures (NNRTI: −3.63 [0.06], n = 11; no NNRTI: −3.69 [0.07], n = 29; *P* = .6; PI: −3.67 [0.09], n = 21; no PI: −3.67 [0.05], n = 19; *P* = 1.0) (Supplementary Figure 2*D*–*I*).

Based on the Frascati classification of NCI (1-way ANOVA *P* = .37), global T-score (*r* = −0.10, n = 30, *P* = .6), GDS (*r* = 0.09, n = 30, *P* = .6), executive function (*r* = 0.23, n = 33, *P* = .20), or any other cognitive domain, no associations were seen between cognitive function and mtDNA CD levels (Figure 2D, Supplementary Figure 2*J–L*).

### Deep Resequencing of mtDNA Point Mutations

Eighty-six samples (48 cases, 38 controls) showed adequate coverage and were included in the analysis. We first examined homoplasmic mtDNA mutations. We observed a median of 30 (interquartile range [IQR], 12.75–34) homoplasmic mutations per sample in HIV-positive cases, which did not differ significantly from HIV-negative controls (median, 23.5 [IQR, 11.75–33]; Mann-Whitney *P* = .3).

**Figure 3.**
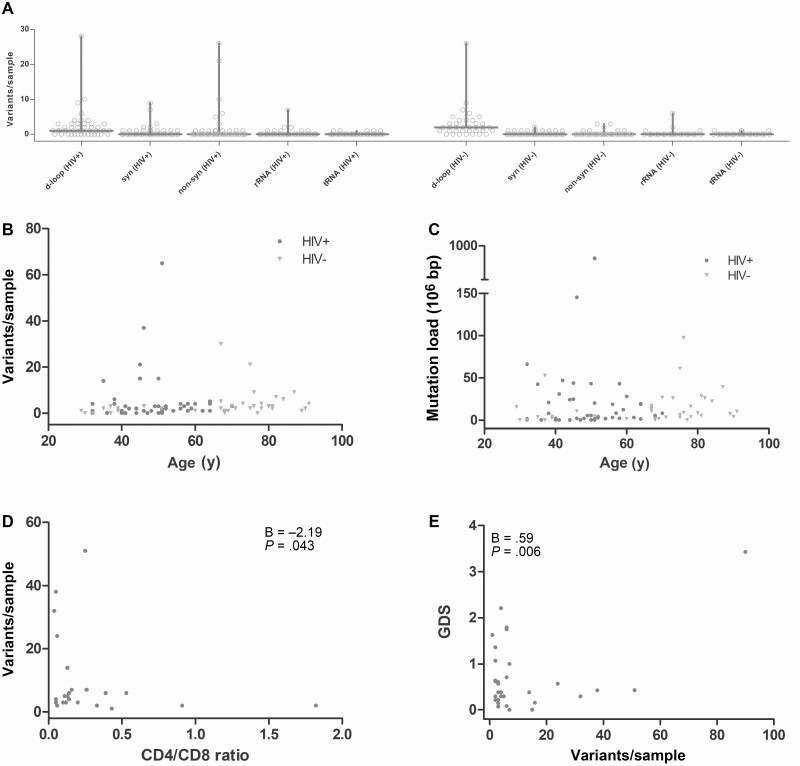
Deep sequencing of heteroplasmic mitochondrial DNA (mtDNA) point mutations (Illumina) in frontal cortex, showing number and type of mutations detected per sample. *A*, Coding region mutations were more frequent in human immunodeficiency virus (HIV)–positive cases compared with controls (horizontal line shows median, bar shows maximum). *B*, Number of mutations increased with age and HIV status. *C*, Mutational burden weighted for variant heteroplasmy levels was higher in HIV-positive cases than controls after adjusting for age. Low CD4/CD8 ratio (*D*) and cognitive impairment (*E*) were associated with increased number of mtDNA mutations. Abbreviations: GDS, global deficit score; HIV, human immunodeficiency virus; non-syn, nonsynonymous; rRNA, ribosomal RNA; syn, synonymous; tRNA, transfer RNA.

We therefore next analyzed heteroplasmic mtDNA point mutations. Total mutation count (expressed here as the number of base positions showing heteroplasmic mtDNA mutations) was a median of 2 per sample (IQR, 1–4) in both HIV-positive cases and HIV-negative controls. Mutations were most commonly seen in the mtDNA noncoding d-loop (2.6 mutations per sample, −2.64 log_10_ per base position), followed by protein-coding genes (1.6 mutations per sample, −3.85 log_10_/bp). Ribosomal RNA (rRNA) mutations (0.3 per sample, −3.92 log_10_/bp) and transfer RNA (tRNA) mutations (0.1 per sample, −4.16 log_10_/bp) were comparatively rare. Number of heteroplasmic mutations per sample was significantly correlated with both age and HIV status (age: regression coefficient [B] = .02, *P* = .029; HIV status: B = .85, *P* = .008; model fit *P* = .029, Figure 3B). We then examined specific mutation types. The number of d-loop mutations was significantly associated with age (*P* = .003) and had borderline significance for HIV status (*P* = .098). The number of coding region mutations was associated with HIV status only (*P* = .002). Neither HIV status nor age was predictive of the numbers of tRNA or rRNA mutations, although these mutation types were rare (Figure 3A).

As mtDNA point mutations present at high heteroplasmy levels are likely to have greater functional effects than those at low levels, we also considered a mutation burden per sample, by weighting mutation counts for their heteroplasmy levels (Supplementary Figure 3*A*). Here we found that heteroplasmy-weighted mutation burden was significantly correlated with HIV status but not with age (age: B = .01, *P* = .16; HIV status: B = .96, *P* < .001; model fit *P* = .002; Figure 3C). This effect of HIV was statistically significant for all mutation types.

We then examined whether HIV- or ART-related factors were associated with mtDNA mutation count in HIV-positive cases. All analyses were adjusted for age. Whereas current CD4 count was not associated with number of mtDNA mutations (*P* = .7), a low CD4/CD8 ratio was significantly associated with an increased number of mutations (B = −2.19 [−4.30 to −0.07], *P* = .043). Interestingly, increasing HIV viral load was associated with decreased number of mutations (B = −0.34 [−0.55 to –0.14], *P* = .001) (Figure 3D, Supplementary Figure 3*B* and 3*C*). Whether a subject was on ART at time of death or whether they had history of exposure to specific NRTIs was not associated with number of mtDNA mutations.

We then examined the relationship between measures of NCI and mtDNA mutation count. Severity of NCI, both global and specific to executive function, was associated with increased mtDNA mutation count (global T-score: B = −.71, *P* = .007; GDS: B = .59, *P* = .006; executive function deficit score: B = 1.01, *P* < .001). This effect was not seen with Frascati classification of NCI (Figure 3E, Supplementary Figure 3*D* and 3*E*).

## Discussion

Here we show for the first time that mtDNA quantity and quality decrease in the brain of PLWH. In all 3 of our measures of mtDNA damage, we saw a significant effect of age at death: Increasing age was associated with an increase in mtDNA mutation load (both point variants and deletions) and a decrease in cellular mtDNA content. These findings are in keeping with the published literature on mtDNA changes in the aging human brain as well as other tissues [7, 30]. Furthermore, we show that HIV infection exacerbates all of these age-associated changes in mtDNA. By a simple analogy, the observed effects of HIV were equivalent to approximately 32-year (for mtDNA content) and approximately 12-year (for mtDNA deletions) age advancement (calculated by expressing the regression coefficient for HIV status relative to the regression coefficient for age).

Most prior data on mtDNA changes in PLWH have suggested that the predominant driver of mtDNA depletion and mutation is ART, and especially the historical NRTIs that inhibit pol γ. In contrast, here we show strong effects of HIV infection per se, but not of ART or of specific NRTIs. What are the likely explanations for these contrasting findings? First, the penetration of ART into the central nervous system (CNS) is variable, but drug levels are always significantly lower than those seen in plasma [31]. As NRTI-induced mitochondrial toxicity is dose-dependent, it is plausible that drug levels achieved in the CNS are below a threshold at which significant mtDNA damage will occur. Another possibility is that the cells of the CNS are more sensitive to the effects of HIV itself on mtDNA compared with many other tissues. Thus, when observing changes in mtDNA, the HIV effect is dominant over any ART effect. How might HIV infection itself result in a reduction in mtDNA quantity and quality in the brain? As neurons have high energy demand, they contain a large number of mitochondria per cell. Thus, neuronal dysfunction and neuronal loss are often associated with changes in mtDNA [32]. In keeping with this hypothesis, in vitro studies have shown that HIV proteins may exhibit direct neurotoxicity mediated by mitochondrial dysfunction [33]. Neurons are likely to be exposed to such viral proteins owing to the productive infection of adjacent glial cells. Interestingly, we saw an association between adverse CD4/CD8 ratio and the number of mtDNA point mutations, plausibly suggesting a legacy effect of advanced HIV in the brain.

The nature of postmortem brain tissue inevitably places some limitations on the generalizability of our findings to healthy PLWH. While we excluded cases with other brain pathology or opportunistic infections, the group as a whole shows advanced HIV (median CD4 count, 70 cells/µL). In a larger and more homogenous set of cases, it would be important to establish whether consistent viral suppression and immune reconstitution are associated with attenuation of the observed changes in mtDNA. We observed associations between mtDNA point mutations and measures of cognitive impairment, although these effects were not seen for mtDNA deletions or depletion. Similarly, it would be important to confirm these findings in a more homogeneous dataset (eg, limited to cases on suppressive cART).

In conclusion, we have shown that PLWH with predominantly advanced disease have abnormalities of mitochondrial DNA quality and quantity in the brain. These changes appear to exacerbate those seen with the normal aging process, and may contribute to NCI. As such, these findings parallel those reported from brain imaging [34]. These observations give weight to the hypothesis that HIV infection may adversely affect biological pathways of aging including the maintenance of mitochondrial function. As these changes were related to HIV infection per se rather than ART, this reinforces the importance of early and effective ART. Future therapies that promote mitochondrial biogenesis may be worthy of evaluation in PLWH.

## Supplementary Data

Supplementary materials are available at *Clinical Infectious Diseases* online. Consisting of data provided by the authors to benefit the reader, the posted materials are not copyedited and are the sole responsibility of the authors, so questions or comments should be addressed to the corresponding author.

ciaa984_suppl_Supplementary_Material

## Notes

***Acknowledgments.*** The authors are grateful for the support of the following resources: Newcastle Brain Tissue Resource, Edinburgh Brain and Tissue Bank, Newcastle University Genomics Core Facility, and Newcastle University Infectious Diseases Facility.

***Financial support.*** This work was supported by Wellcome (grant numbers 109975/Z/15/Z, 203105/Z/16/Z) and by shared funding through the National Institute of Mental Health and the National Institute of Neurological Disorders and Stroke (Manhattan HIV Brain Bank, grant number U24MH100931; Texas NeuroAIDS Research Center, grant number U24MH100930; National Neurological AIDS Bank, grant number U24MH100929; California NeuroAIDS Tissue network, grant number U24MH100928; Data Coordinating Center, grant number U24MH100925). B. A. I. P. reports an infrastructure grant from the Wellcome Centre for Mitochondrial Research, outside the submitted work.

***Potential conflicts of interest.*** The authors: No reported conflicts of interest. All authors have submitted the ICMJE Form for Disclosure of Potential Conflicts of Interest.
