# Supplementary material for: Mitochondrial DNA Damage and Brain Aging in Human Immunodeficiency Virus
Source: Clin Infect Dis. 2020 Jul 28;73(2):e466–73. doi: 10.1093/cid/ciaa984 (PMC8282328; doi:10.1093/cid/ciaa984)

Supplementary material

Supplementary methods

Table S1. Oligonucleotides

	Forward primer (5'-3')	Reverse primer (5'-3')	Probe (5'-3')
qPCR *B2M*	CACTGAAAAAGATGAGTATGCC	AACATTCCCTGACAATCCC	FAM-CCGTGTGAACCATGTGACTTTGTC-BHQ1
qPCR *MT-ND1*	ACGCCATAAAACTCTTCACCAAAG	GGGTTCATAGTAGAAGAGCGATGG	HEX-ACCCGCCACATCTACCATCACCCTC-BHQ1
qPCR CD	CCCACCATAATTACCCCCATAC	GGAGTAGAAACCTGTGAGGAAAGG	Cy5-CCTACCTCCCTCACCATTGG-BHQ2
qPCR standard *B2M*	CGCAATCTCCAGTGACAGAA	GCAGAATAGGCTGCTGTTCC	-
qPCR standard *MT-ND1*	CAGCCGCTATTAAAGGTTCG	AGAGTGCGTCATAGTTGTTC	-
qPCR standard CD	TCCTAACACTCACAACAAAAC	GTTAGGTAGTTGAGGTCTAGG	-
LR-PCR fragment 1	CCCTCTCTCCTACTCCTG	CAGGTGGTCAAGTATTTATGG	-
LR-PCR fragment 2	CATCTTGCCCTTCATTATTGC	GGCAGGATAGTTCAGACG	-

Supplementary figures

Figure S1. Relationship between (age-corrected) mtDNA content and clinical parameters in HIV positive cases. (A) CD4 count, (B) HIV-1 plasma viral load, (C) CD4/CD8 ratio, (D) didanosine (ddI) exposure, (E) zalcitabine (ddC) exposure, (F) stavudine (d4T) exposure, (G) zidovudine (AZT) exposure, (H) protease inhibitor (PI) treatment, (I) non-nucleoside reverse transcriptase inhibitor (NNRTI) treatment, (J) neurocognitive impairment (Frascati classification), (K) global T-score [dotted lines are normal, 50, and SD, 10], (L) executive function deficit score.

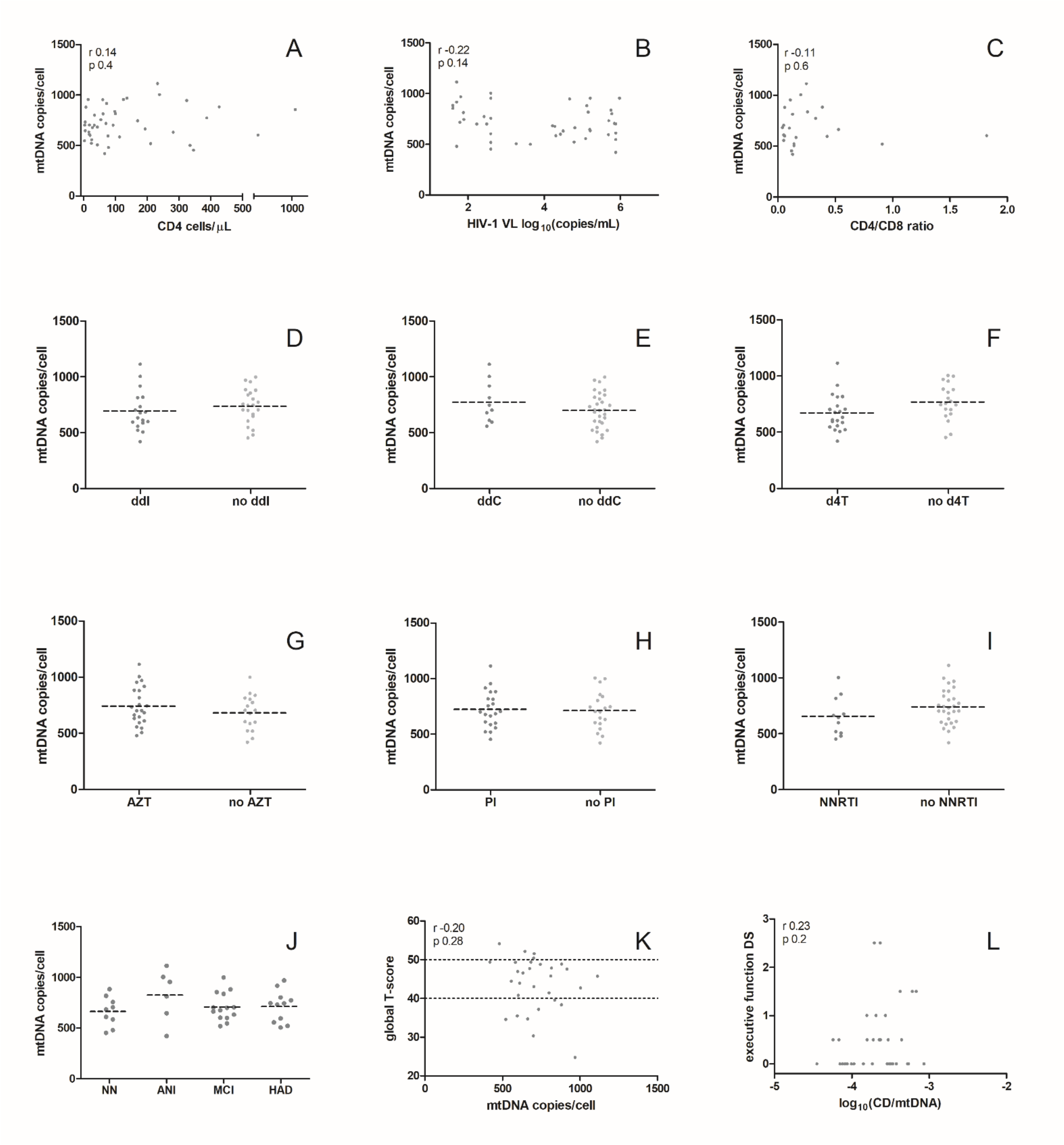

Figure S2. Relationship between (age-corrected) mtDNA common deletion (CD) levels and clinical parameters in HIV positive cases. (A) CD4 count, (B) HIV-1 plasma viral load, (C) CD4/CD8 ratio, (D) didanosine (ddI) exposure, (E) zalcitabine (ddC) exposure, (F) stavudine (d4T) exposure, (G) zidovudine (AZT) exposure, (H) protease inhibitor (PI) treatment, (I) non-nucleoside reverse transcriptase inhibitor (NNRTI) treatment, (J) neurocognitive impairment (Frascati classification), (K) global T-score, (L) executive function deficit score.

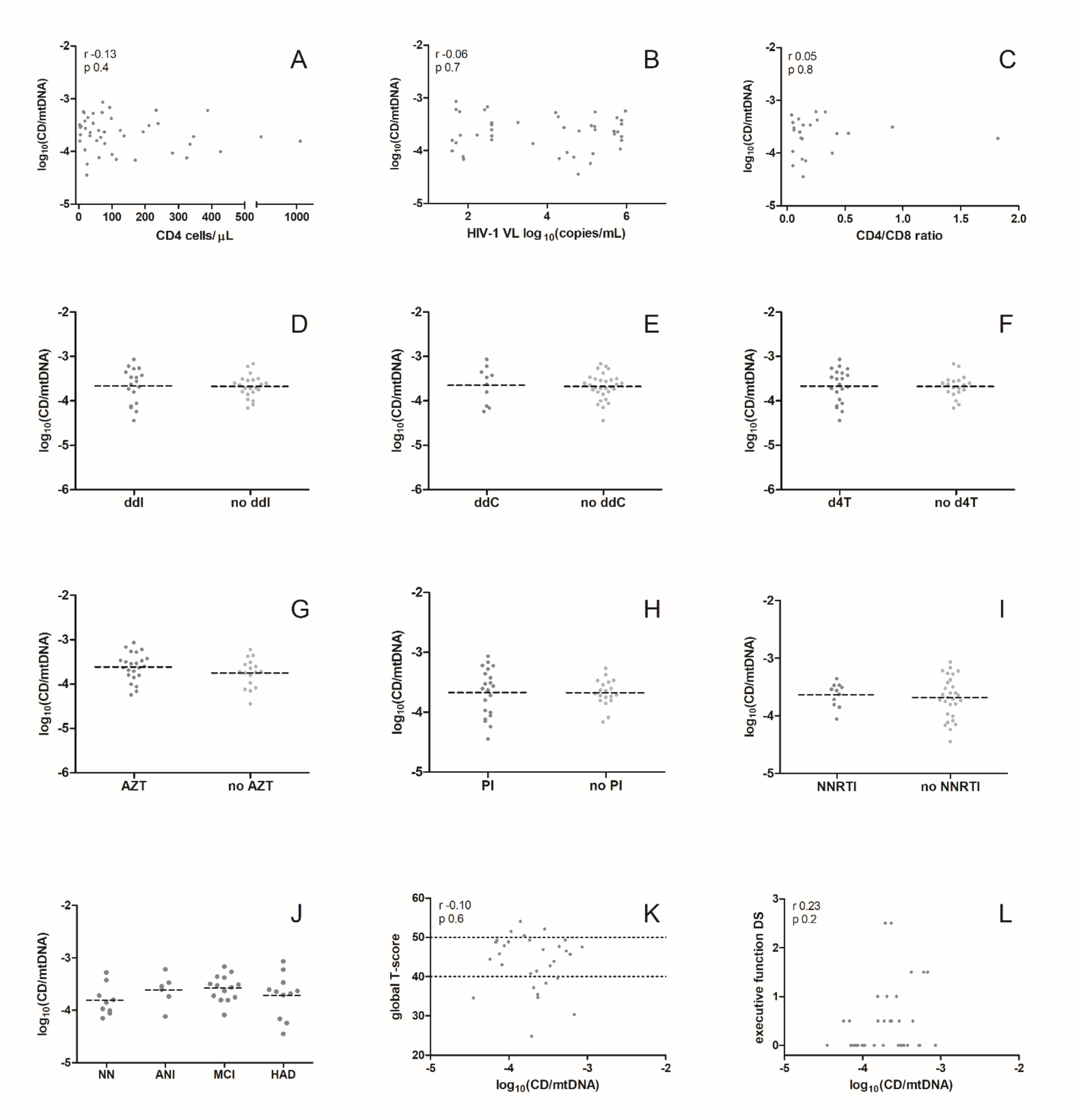

Figure S3. Deep sequencing of heteroplasmic mtDNA point mutations (Illumina). (A) Observed heteroplasmy levels of variants according to variant type and HIV status. Each dot represents an individual variant. (B-E) Relationship between clinical parameters and number of point mutations: (B) CD4 count, (C) HIV-1 plasma viral load, (D) global T-score, (E) executive function deficit score.

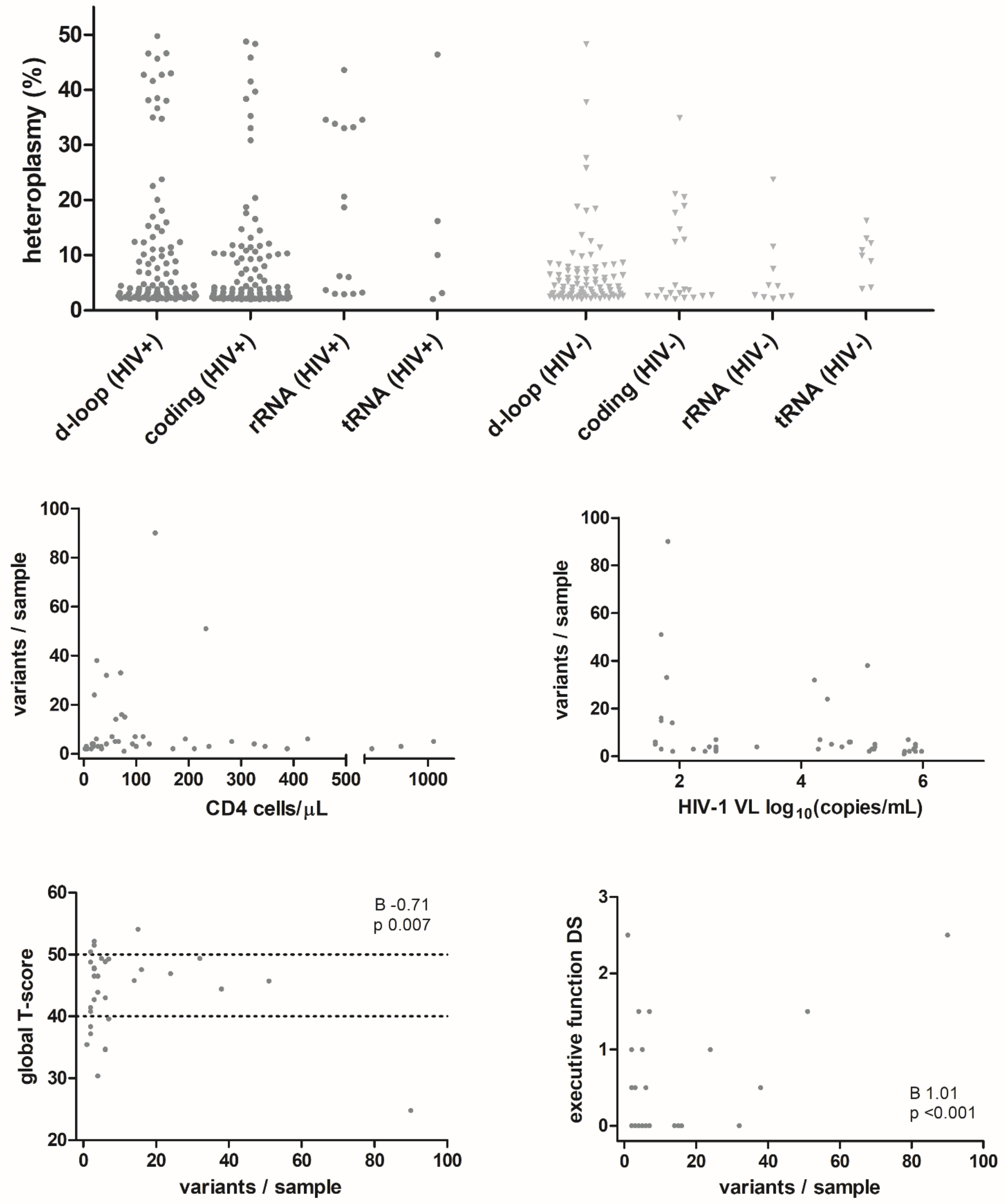

Supplement: ciaa984_suppl_Supplementary_Material [file ciaa984_suppl_supplementary_material.pdf]
